# The Role of the **γ** **δ** T Cell in Allergic Diseases

**DOI:** 10.1155/2014/963484

**Published:** 2014-06-03

**Authors:** Rui Zheng, Qintai Yang

**Affiliations:** Department of Otorhinolaryngology-Head and Neck Surgery, The Third Affiliated Hospital, SUN Yat-sen University, Guangzhou 510630, China

## Abstract

The predominant distribution of **γ** **δ** T cells in the mucosal and epithelial tissues makes these unconventional lymphocytes the “guards” to contact external environment (like allergens) and to contribute to immune surveillance, as well as “vanguards” to participate in initiating mucosal inflammation. Therefore, **γ** **δ** T cells have been considered to bridge the innate and adaptive immunity. The role these cells play in allergy seems to be complicated and meaningful, so it makes sense to review the characteristics and role of **γ** **δ** T cells in allergic diseases.

## 1. Introduction

*γδ* T cells are a minor population of lymphocytes expressing *γ* and *δ* T cell receptor (TCR) chains, which are often considered to bridge innate and adaptive immune responses. Recent studies have shown that *γδ* T cells can comprise up to 50% of the T cells within epithelium or mucosa-rich tissues and less than 10% in peripheral blood [1]. The specific localization and abundance of these cells suggest that they might be markedly implicated in epithelial/mucosal immunity [2, 3]. In contrast to recognition of antigens by *αβ* T cells, *γδ* T cells recognize antigens directly without any requirement for antigen processing and presentation or major histocompatibility complex (MHC) molecules [4]. It has been indicated that *γδ* T cells may play crucial roles in the development and perpetuation of allergic inflammation as effector and immunoregulatory cells, via production of T helper (Th)1-, Th2-, and Th17-associated cytokines [5], which not only induce the synthesis of IgE but also recruit effector cells like eosinophils and basophils into the site of allergic inflammation [6]. Besides, different subsets of *γδ* T cells can show different functions, depending on what tissue they are found in and which specific TCRs they bear [7]. Even though there is a growing consensus about the importance of these cells in allergic immune responses, the specific mechanisms remain elusive. The present review focuses on the latest knowledge on characteristics and role of *γδ* T cells in allergic diseases.

## 2. **γ** **δ** T Cells Have Diverse Subsets with Specific Locations and Functions

As research continues, it has been realized that *γδ* T cells are not a homogeneous population of cells with a single physiological role, and their subset complexity is being characterized, both in mice and humans [7]. TCR V*γ*- and V*δ*-encoded chain pairs may interact with distinct ligands in different tissues and be expanded on that basis. Defined by the usage of either V*δ*1 or V*δ*2 TCR (V*δ*3 and V*δ*5 making up minor populations), human *γδ* T cells fall into two major subsets: V*δ*2 T cells account for the majority (50–95%) of circulating *γδ* T cells, whereas V*δ*1 T cells are rare in the blood but appear at increased frequencies in mucosal tissues and in the skin [7–10].

The V*γ*9V*δ*2 (also termed V*γ*2V*δ*2, collectively designated V*δ*2) T cells in the peripheral blood can sometimes identify over 50% of leucocytes after certain bacterial or parasitic infections and rapidly get activated; therefore, such TCR-dependent activation of V*γ*9V*δ*2 T cells enables them to respond to a diverse range of pathogens [11]. According to the surface expression of CD45RA and CD27, markers more commonly used to identify the naive, effector, or memory status of conventional *γδ* T cells, human V*γ*9V*δ*2 T cells, are often subdivided into four subsets: “naive” (Tnaive) CD45RA^+^CD27^+^ cells; “central memory” (TCM) CD45RA^−^CD27^+^ cells; “effector memory” (TEM) CD45RA^−^CD27^−^ cells; “CD45RA^+^ effector memory” (TEMRA) CD45RA^+^CD27^−^ cells [7, 12]. Tnaive and TCM cells express lymph node homing receptors, abound in lymph nodes, and lack immediate effector functions. Conversely, TEM and TEMRA cells, which express receptors for homing to inflamed tissues, are poorly represented in the lymph nodes while abounding in sites of inflammation and display immediate effector functions. It indicates a lineage differentiation pattern for human V*δ*2 T cells that generates naive cells circulating in lymph nodes, effector/memory cells patrolling the blood, and terminally differentiated effector cells residing in inflamed tissues [12].

In contrast to V*δ*2, the TCR-*γ* chain usage by the tissue-associated V*δ*1 T cells varies at distinct anatomic locations. V*δ*1 T cells in the periphery express a naive phenotype and may migrate preferentially to localized sites when they are activated [13]. For instance, V*γ*2, V*γ*3, V*γ*5, V*γ*6, and V*γ*7 are used predominantly by *γδ* T cells in peripheral lymphoid organs, skin, small intestine, tongue, and reproductive system, respectively [14, 15]. In contrast, V*γ*1 and V*γ*4 are preferentially expressed in the respiratory system like nasal mucosa and lung [16–18]. Recent studies have indicated that these tissue-associated V*δ*1 T cells may play an important function not only in maintaining immune homeostasis in the local microenvironment [19] but also in wound healing, removing distressed or transformed epithelial cells and subduing excessive inflammation, in both mice and humans [20–22]. Besides, the role of these mucosa predominantly expressed *γδ* T cells in allergic diseases has also been noticed. Our preliminary studies found that the infiltration of *γδ* T cells significantly increases in the nasal mucosa of patients with perennial allergic rhinitis (AR) (data not shown). Moreover, Pawankar et al. [23] proved that the increased population of *γδ* T cells in the perennial AR patients' nasal mucosa mainly comprises of V*γ*1V*δ*1 subsets. In mice sensitized and challenged with OVA, Cook et al. [24] observed that V*γ*1^+^ T cells spontaneously enhance airway hyperresponsiveness (AHR), whereas V*γ*4^+^ T cells, after being induced by allergen sensitization and challenge, suppress AHR. These data suggest that *γδ* T cells of distinct phenotypes may play different, sometimes opposed, functions in airway allergic inflammation. However, it is still premature to speculate whether the V*γ*4^+^ subsets of *γδ* T cells may exert an important role in maintaining immune homeostasis in local microenvironment of healthy humans; on the other hand, the V*γ*1^+^ subsets may take an essential part in the development and perpetuation of allergic inflammation as effectors in atopy patients. Besides, our another recent study showed that different subsets of *γδ* T cells in peripheral blood of perennial AR patients before and after specific immunotherapy (SIT) appear with distinct expression patterns [25]. But whether *γδ* T cells in peripheral blood and in mucosa function separately or synergistically remains an unsolved problem.

## 3. **γ** **δ** T Cells in Different Age and Gender Groups

With age, there comes the change from having fairly diverse pairs of *γδ* T cells (of which V*δ*1^+^ subsets serve as the majority in cord blood at birth) to increasingly restricted pairings (with V*γ*9V*δ*2 T cells becoming the major subsets with very limited receptor diversity by adulthood). From birth to about 10 years of age, the absolute number of *γδ* T cells in the periphery increases, with the V*γ*9V*δ*2 T cell subsets expanding from a minor population at birth to usually more than 75% of circulating *γδ* T cells [13, 26]. V*γ*9V*δ*2 T cells are known to respond to many different phosphoantigens, so it is likely that the exposure to a variety of pathogens results in the selection of these cells in early life [27]. This clonal expansion has been seen as evidence of the vital role these T cells play in responding to environmental challenges in early life. However, it has not been demonstrated whether this phenomenon is in fact primary in response to environmental challenges but not, at least in part, endogenous stimuli, as an extension of the adaptive changes taking place within the newborn [10].

Previous longitudinal cohort studies have shown that most childhood asthma begins in infancy, and between 40% and 75% of children with asthma will have complete resolution of symptoms by adolescence or adulthood [28]. Respiratory syncytial virus (RSV) infection in lower respiratory tract in early childhood is a risk factor for the subsequent development of allergic sensitization such as wheezing up to age of 11 years [29]. Aoyagi et al. [30] reported that compared to age-matched controls, infants affected by RSV-bronchiolitis have lower frequencies of IFN-*γ*-producing *γδ* T cells in peripheral blood. Moreover, they noticed normalization of this frequency during the convalescent phase, suggesting that the defective IFN-*γ* production by these cells may play an important role in the development of asthma. However, it is too early to conclude whether the expansion of V*δ*2V*γ*9 T cells with age is associated with childhood asthma spontaneous remission by adolescence.

In adulthood, studies have also found the possible great impact of age and gender on the *γδ* T cell repertoires: in contrast to childhood, the absolute number of *γδ* T cells decreases, as the result of reduction of V*δ*2, but not V*δ*1 T cells. Besides, the number of total *γδ* T cells and V*δ*2 T cells are both significantly higher in males than in females [31, 32]. It indicates that age- and gender-matched controls are essential for clinical studies of *γδ* T cell repertoires in patients.

The term “allergic march” refers to the natural history of atopic manifestations, which is characterized by a typical sequence of IgE antibody responses and clinical symptoms that appear early in life, persist over years or decades, and often remit spontaneously with age. Several studies have shown that the “new” allergy can occur throughout life; generally, allergy prevalence and severity tend to decrease after young adult life [33], and Th2-type responses may weaken with age [34]. Hansen et al. [35] found that immunization dose, sex, and age are highly influential on allergy outcomes in murine models. Nevertheless, further researches are required to make certain whether the change of *γδ* T cell subsets is associated with the age and gender related allergic march.

## 4. The Antigen Recognition of **γ** **δ** T Cells in Allergy

Selective allergen recognition by TCR that binds specific regions of the antigen molecules is the priming and initiation of antigen-specific T cell immune responses. So far, more than 4000 substances in the environment, the vast majority of which are proteins, mostly enzymes, have been identified as allergens that elicit an IgE-mediated immune response in a genetically predisposed individual. Different from MHC-restricted recognition of bound peptides by *αβ* TCRs, the antigen specificity of *γδ* T cells involves the immunoglobulin-like structure of the *γδ* TCR [36] with the recognition of unprocessed peptides, small organic phosphate molecules, or alkylamines derived from microbes and edible plants [37].

Studies have shown that the epithelial-associated *γδ* T cells can recognize stress-induced self-antigens, which enables them to monitor multiple insults to the epithelium [38]. However, data from humans and mice seem to indicate the relevance of mucosa-associated *γδ* T cells in allergen recognition and airway inflammation, perhaps mediated by interaction of foreign antigens with CD1^+^ dendritic cells (DCs) [39]. The study by Russano et al. [40] showed that CD1^+^ immature DCs expand in the respiratory mucosa of allergic subjects and are able to process both proteins and lipids, and CD1-restricted phospholipids (PL)-specific *γδ* T cells represent the key mucosal regulatory subsets for the control of early host reactivity against tree pollens. These CD1-restricted *γδ* T cells can respond promptly to lipid-antigen recognition by secreting a wide array of cytokines, including high amounts of IL-4, and expand at mucosal allergic inflammation sites [38]. In addition, *γδ* T cells derived from nasal mucosa in allergic subjects could also recognize pollen derived PE in a CD1d-restricted fashion [40]. Therefore, it may be speculated from above that the early allergic response initiates with primary mucosal recognition of allergen by CD1^+^ DCs and CD1-restricted *γδ* T cells, which ensure rapid handling of the foreign inhaled grain.

## 5. The Cytokine Production of **γ** **δ** T Cells in Allergy

It is proposed that *γδ* T cells may act as an extended arm of *αβ*T cells, by providing a rapid but weaker response before the *αβ* T-cell response has fully developed. Substantial evidence has been accumulated to indicate that *γδ* T cells have the potential to produce Th17-type cytokines (like IL-17) and Th2-type cytokines (IL-4, -5, and -13) and thus enhance airway allergic inflammation and AHR [41]. In contrast, Th1-type cytokines (IL-12 and IFN-*γ*) produced by *γδ* T cells might be induced after special immunotherapy and inhibition of these allergic diseases [42, 43].

The study by Ribot et al. [44] showed that in the spleen and lymph nodes and in the peripheral tissues of mice, the presence or absence of the cytokine CD27 distinguishes two *γδ* T cell subsets: the CD27^+^ cells produce IFN-*γ*, whereas the CD27^−^ cells (50% of V*γ*4^+^ and 11% of V*γ*1^+^) produce primarily IL-17. It suggests the existence of a differential requirement for optimum activation of these distinct *γδ* T cell subsets in the peripheral immune compartment. In humans, 80% of circulating V*γ*9V*δ*2 T cells are IFN-*γ* producers, while less than 1% produce IL-17 [45]. Caccamo et al. [46] observed that IFN-*γ* ^+^ V*γ*9V*δ*2 T cells have a predominant TEM and at a lower extent TEMRA phenotype, while IL-17^+^ V*γ*9V*δ*2 T cells exhibit a TEMRA phenotype. However, IL-17^+^ V*γ*9V*δ*2 T cells significantly increase and secrete abundant IL-17 at sites of inflammation (perhaps primarily at epithelial surfaces), which may directly shape the inflammatory infiltrate, for example, by attracting neutrophils during bacterial infection [45]. Zhao et al. [47] also found that the IFN-*γ* ^+^/IL-17^+^ ratio in *γδ* T cells significantly decreases in patients with allergic asthma compared with healthy controls. What is more, several *γδ* T cells are indicated to be a chief source of IL-17 in peripheral tissues such as lung, which share certain common features with Th17 cells [48–50]. With regard to humans, *γδ* T cells are divided into two different phenotypes. An IL-4-producing phenotype, which possesses V*γ*1/V*δ*1 segments, enhances allergic inflammation. V*γ*9/V*δ*2 segments have an IFN-*γ*-producing phenotype and might thus have a partial ability to modulate allergen-specific Th2-skewed immunity [43].

In a word, after quick antigen recognition and full activation, diverse subsets of *γδ* T cells in the circulating blood and various lymphoid compartments subsequently produce an array of different cytokines, performing proinflammation as well as pleiotropic immunoregulatory functions, as it will be further discussed.

## 6. **γ** **δ** T Cells Are Both Effector and Regulatory Cells in Allergic Inflammation

**Figure 1:**
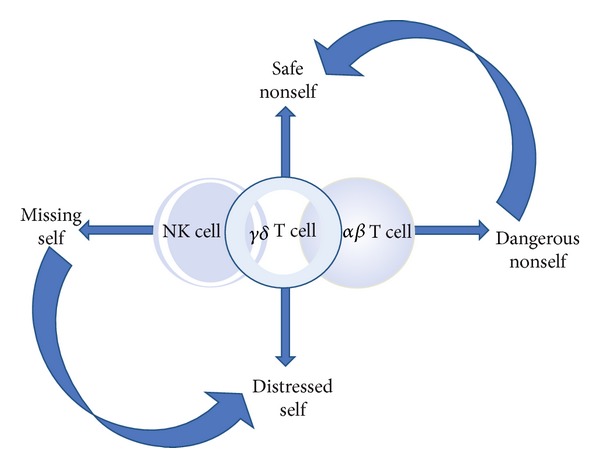
A simplified paradigm illustrating where in the continuum of immune protection and homeostasis *γδ* T cells fall in relation to innate NK cells and the adaptive *αβ*T cells. Innate NK and adaptive *αβ* T cells respond to the “missing self” and the “dangerous nonself,” respectively, while, between these two extremes, *γδ* T cells respond to the “safe nonself” and deal with the inevitable “distressed self.” These different “selves” and the immune response(s) that they trigger exist in a continuum and are modulated by the context in which they are presented. Besides, NK cells could contribute to responding to the “distressed self,” whereas *αβ* T cells have some regulatory training to temper the response to the “safe nonself” (cited from [10]).

Lately, Kalyan and Kabelitz [10] provided a biographical sketch of *γδ* T cells: in a continuum, innate natural killer (NK) cells and adaptive *αβ* T cells respond to the “missing self” and the “dangerous nonself,” respectively, while *γδ* T cells respond to the “safe nonself” and deal with the inevitable “distressed self.” What is more, NK cells could contribute to responding to the “distressed self,” whereas *αβ* T cells have some regulatory training to temper the response to the “safe nonself” [10] (Figure 1). This sketch supports the viewpoint that *γδ* T cells serve as the bridge between innate and adaptive immunity. Allergy is primarily considered as a classic Th2-driven immune response against allergens (safe nonself), with important contributions to pathology by Th2-type cytokines IL-4, -5, and -13, which not only induce the synthesis of IgE but also recruit effector cells like eosinophils and basophils into the site of allergic inflammation [42]. However, inflammatory responses in allergic diseases are more complex than simple overexpression of Th2 cytokines. A recent hypothesis has been put forward to rely on the genetically determined barrier deficiency and disruption by environmental and endogenous proteases in the epithelial barrier (distressed self), which might result in the allergen uptake as a primary defect in the pathogenesis of allergic reactions [51]. It seems that allergy is both an epithelial disease and a disease of the immune system. Using an adoptive cell transfer approach, Jin et al. [52] found that NK and *γδ* T cells (only V*γ*1V*δ*5 subsets) are necessary for the acute stages of AHR in mice but not for the later airway eosinophilic inflammation. Another study on AHR demonstrated that NK cells secreted IL-4 and -13 to produce their effector function, but *γδ* T cells did not have this effect [53]. In addition, *γδ* T cells, similar to NK cells, express the NKG2D receptor that may contribute to effective stress-responses as well as immune surveillance, which may be relevant in the induction of food allergy [54]. These data suggest that the interaction of innate and adaptive immune cells and the impact of the inflammatory responses on this collaboration seem to be important and worthy of further research.

Abundant populations of *γδ* T cells have been found in the epidermis of rodents [55]. The respiratory mucosa such as nasal mucosa, bronchial mucosa, and lung contain *γδ* T cells as well [56]. This may be of importance in the remarkable resistance of the airway against environmental stimuli. Substantial evidence has been accumulated to indicate that *γδ* T cells take part in Th2 immune responses. *γδ* T cells themselves can not only take the function of follicular Th cells in certain responses but also can support responses that are dependent on classical help provided by *αβ* T cells. An increase in *γδ* T cells expressing Th2-type cytokines has been reported in bronchoalveolar lavage (BAL) fluids of allergen challenged asthmatic patients [57]. In addition to proinflammatory function, the *γδ* T cells also engage as regulators of Th2 immunity [41], in particular regulating the IgE antibodies [3, 38, 40]. Svensson et al. [58] showed that *γδ* T cell-deficient mice exhibited a diminished allergen specific IgE response compared with wild-type (WT) mice, indicating that *γδ* T cells contribute to B cell secretion of allergen-specific IgE, either by promoting Ig class switch to IgE or by providing activation signals to differentiated IgE-producing cells. Likewise, Zuany-Amorim et al. [59] reported a low antigen specific IgE and IL-5 release and a decrease in T cell infiltration in the same mouse models. They further found that the response could be restored when IL-4 was administered, suggesting that *γδ* T cells contribute to type 2-mediated airway inflammation by inducing IL-4 dependent IgE and IgG1 responses. In contrast, Lahn et al. [60] showed that *γδ* T cells exert a suppressive role in the Th2 response to allergen challenge. Therefore, it is clear that *γδ* T cells might have various, possibly opposing roles for CD4^+^ T cells.

Studies have demonstrated that, in the airway, distinct subsets of *γδ* T cells, defined by their expression of TCR-*γ*, seem to exhibit differential and sometimes opposed Th-like reactivities in allergen-induced allergic inflammation [7, 47]. In mouse models of allergic diseases, it has been shown that the V*γ*1^+^ subsets can enhance AHR as well as levels of Th2 cytokines in the airways and eosinophilic infiltrates in the lungs [61, 62], and, in contrast, the V*γ*4^+^ subset can be induced to inhibit AHR [63, 64]. Lahn et al. [64] selectively depleted either subset in the lungs (using aerosolized, inhaled anti-TCR Abs) following airway challenge and observed that AHR is altered in the predicted fashion; that is, depletion of V*γ*1^+^ cells decreases and depletion of V*γ*4^+^ cells increases AHR. After transferring few purified V*γ*1^+^ cells into OVA/alum immunized TCR-*δ* ^−/−^ mice, Huang et al. [65] observed the increase of the OVA-specific IgE responses, suggesting that individual enhancer cells are quite potent. However, the relative importance of *γδ* T cells in human asthma remains to be determined.

It has been shown for some time that murine *γδ* T cells become functionally competent in the thymus, particularly regarding the production of proinflammatory cytokines IFN-*γ* and IL-17 [60]. McMenamin et al. [66] found that *γδ* T cells regulate IgE responsiveness to inhaled antigens by high production of IFN-*γ*. By using mice immunized with recombinant vaccine virus expressing RSV F protein and challenged with live RSV, Dodd et al. [63] reported that V*γ*4^+^ subsets are recruited into the lungs and produce IFN-*γ* in a time-dependent manner. These studies suggest that antigen-specific *γδ* T cells are able to suppress the pathogenic Th2 response in allergic asthma, whereas a recent study by Chen et al. [67] found that the serum levels of IL-4 and IL-13 in peripheral blood of children with AR and asthma markedly decrease while IFN-*γ* increases after receiving SIT, suggesting that IFN-*γ* ^+^ *γδ* T cells might exert their Th2 immunosuppression under certain conditions like SIT. Our recent study showed that the serum levels of IL-17 and IL-23 in the AR patients were significantly higher than those in the healthy subjects, and positive correlations exist between the IL-17 and the IL-23 levels, as well as the IL-17 level and *γδ* T frequencies [68]. Accordingly, we conjecture that the IL-23R^+^ IL-17^+^ *γδ* T cells may promote *αβ* T cell-mediated traditional Th2 inflammation via producing abundant IL-17. In addition, IL-17-producing *γδ* T cells could directly promote the development of other IL-17-producing T cells [69], and these innate IL-17-producing T cells are involved in sensing stress, injury, or pathogens and serve an immunoregulatory role at epithelial sites [63].

Gonçalves-Sousa et al. [70] reported that murine CD4^+^CD25^+^Foxp3^+^ regulatory T cells (Treg) abolish key effector functions and proliferation of *γδ* T cells both in vitro and in vivo. They further showed that the suppression is dependent on cellular contact between Treg and *γδ* T cells and is partially mediated by glucocorticoid-induced TNF receptor-related proteins. It reveals a novel mechanism, by which *γδ* T-cell function is regulated, and suggests that endogenous Treg may prevent the desired effects of *γδ* T cell-based immunotherapies. It has also been shown by Hahn et al. [71] that *γδ* T cells affect the level of IL-10 in the airways and block their function resulting in an increase of Treg in the lung, which suggests that *γδ* T cells might inhibit Treg function. While these data highlight the importance of understanding how the proinflammatory and immunoregulatory functions of *γδ* T cells are regulated, the detailed processes remain poorly understood.

## 7. **γ** **δ** T Cells with the Prevention and Treatment of Allergic Diseases

*γδ* T cells stimulated with bisphosphonate compounds, which are clinically well tolerated and used for *γδ* T cell expanders in vitro and in vivo, have been considered to be good candidates for cancer immunotherapy, because of their IFN-*γ* production and cytotoxic effect [72]. Therefore, the adoptive transfer of autologous *γδ* T cells expanded in vitro might also be an effective strategy for IL-4-mediated allergy.

It has been shown that oral tolerance, which refers to the active state of nonresponsiveness to food and food protein intake, is a unique feature of the (gut-associated) mucosal immune system. And the defects in this process result in allergic sensitization to food proteins [73]. Most intestinal epithelial lymphocytes (IELs) in the mouse consist of *γδ* T cells, which are localized in the paracellular space between intestinal epithelial cells at the luminal site of the basement membrane [74]. Mengel et al. [75] showed that treating mice with a TCR-*δ*-specific antibody results in impaired oral tolerance induction and that oral tolerance could be transferred by means of *γδ* T cells. It suggests that targeting intestinal *γδ* T cells may provide preventing and therapeutic strategies for food allergy. However, currently IELs are among the least studied cells in the process of allergic sensitization.

Studies have shown that many phosphoantigens and fungal immunomodulators play an important role in *γδ* T cell-mediated immunotherapy. For example, (E)-4-hydroxy-3-methyl-but-2-enyl pyrophosphate is characterized as a very potent agonist of V*γ*9V*δ*2 responses and could strengthen the principle of *γδ* T cell based immunotherapy [76]. In contrast, Gonçalves-Sousa et al. [70] described that Treg could negatively modulate the *γδ* T cell activities and stressed the importance of combining Treg inhibition with *γδ* T cell activation for future immunotherapeutic strategies.

In animal models, chronic allergen challenge induces suppression of the Th2 response and reduces AHR and airway inflammation [77, 78]. Reductions of late-phase asthmatic responses to allergen after long-term allergen challenge have also been reported in clinical studies [79, 80]. Lahn et al. [64] indicated that the V*γ*4^+^ subsets appear to mediate such suppressive effect of long-term allergen challenge on AHR. In addition, *γδ* T cells could also suppress Th2-dependent IgE responses without affecting parallel IgG responses to inhaled antigens [66].

Taken together, *γδ* T cells may have the potential to help alter the Th2-skewed immunity in patients with allergic diseases. Further accumulated studies to clarify the ability of *γδ* T cells as an allergic immunotherapy candidate are thus called for.

## 8. Concluding Remarks

There is ample evidence that *γδ* T cells are involved in allergy. Recent studies in humans and mice suggest that they can both drive and regulate allergic immune responses through different mechanisms. However, many aspects of the characteristics and role of *γδ* T cells in allergy remain to be fully elucidated in near future, for instance, the exact effects of various *γδ* T cell subsets on allergic inflammation; the underlying relations between blood- and mucosa-associated *γδ* T cells; how age and gender influence the population, distribution, and function of *γδ* T cells in allergic diseases; the specific regulatory mechanisms of *γδ* T cells in allergy; how *γδ* T cells could be applied to prevent and treat allergic diseases, and so on.
